# Deep Learning for Bone Mineral Density and T-Score Prediction from Chest X-rays: A Multicenter Study

**DOI:** 10.3390/biomedicines10092323

**Published:** 2022-09-19

**Authors:** Yoichi Sato, Norio Yamamoto, Naoya Inagaki, Yusuke Iesaki, Takamune Asamoto, Tomohiro Suzuki, Shunsuke Takahara

**Affiliations:** 1 Department of Orthopedics Surgery, Japan Community Healthcare Organization (JCHO) Tokyo Shinjuku Medical Center, Tokyo 162-8543, Japan; 2 Department of Orthopedics Surgery, Nagoya University Graduate School of Medicine, Nagoya 464-8550, Japan; 3 Department of Orthopedics Surgery, Gamagori City Hospital, Gamagori 443-8501, Japan; 4 Department of Orthopedics Surgery, Miyamoto Orthopaedic Hospital, Okayama 703-8236, Japan; 5 Department of Epidemiology, Graduate School of Medicine, Dentistry and Pharmaceutical Sciences, Okayama University, Okayama 700-8558, Japan; 6 Systematic Review Workshop Peer Support Group (SRWS-PSG), Osaka 541-0043, Japan; 7 Department of Orthopedics Surgery, The Jikei University Kashiwa Hospital, Chiba 277-8567, Japan; 8 Department of Orthopedics Surgery, The National Hospital Organization Nagoya Medical Center, Nagoya 460-0001, Japan; 9 iSurgery Co., Ltd., Tokyo 103-0012, Japan; 10 Department of Orthopaedics Surgery, Hyogo Prefectural Kakogawa Medical Center, Kakogawa 675-0003, Japan

**Keywords:** osteoporosis, screening, DXA, BMD, chest X-ray, deep learning, artificial intelligence

## Abstract

Although the number of patients with osteoporosis is increasing worldwide, diagnosis and treatment are presently inadequate. In this study, we developed a deep learning model to predict bone mineral density (BMD) and T-score from chest X-rays, which are one of the most common, easily accessible, and low-cost medical imaging examination methods. The dataset used in this study contained patients who underwent dual-energy X-ray absorptiometry (DXA) and chest radiography at six hospitals between 2010 and 2021. We trained the deep learning model through ensemble learning of chest X-rays, age, and sex to predict BMD using regression and T-score for multiclass classification. We assessed the following two metrics to evaluate the performance of the deep learning model: (1) correlation between the predicted and true BMDs and (2) consistency in the T-score between the predicted class and true class. The correlation coefficients for BMD prediction were hip = 0.75 and lumbar spine = 0.63. The areas under the curves for the T-score predictions of normal, osteopenia, and osteoporosis diagnoses were 0.89, 0.70, and 0.84, respectively. These results suggest that the proposed deep learning model may be suitable for screening patients with osteoporosis by predicting BMD and T-score from chest X-rays.

## 1. Introduction

With the population aging and increasing life expectancy, osteoporosis has become a global health issue affecting more than 200 million people worldwide [1]. It is the greatest risk factor for fragility fractures such as vertebral and hip fractures, and affects life prognosis [2,3,4]. Early diagnosis of osteoporosis through screening is important for the initiation of therapeutic agents and prevention of fragility fractures [5]. The standard examination for osteoporosis screening is the measurement of bone mineral density (BMD) using dual-energy X-ray absorptiometry (DXA) [6]. However, DXA has drawbacks in terms of high equipment cost and radiation exposure [7,8,9]. Meanwhile, increasing awareness of osteoporosis may be the most effective strategy for the prevention of osteoporotic fractures [10]. However, awareness of this disease among the elderly is very low [11]; therefore, the osteoporosis screening rate in Japan is only 5% [12]. Solutions to these challenges would include (1) using commonly available imaging equipment and (2) using multipurpose imaging equipment that is frequently used in clinical settings. Presently, the most frequently performed imaging technique is chest radiography. A previous study demonstrated that values obtained by analyzing structural and anatomical phenotypes, such as the cortical thickness of the clavicles, ribs, and spine in chest radiographs, are correlated with BMD [13,14,15]. These findings suggest that a tool for predicting BMD from chest X-rays obtained for various medical purposes would be useful for osteoporosis screening. The utilization of chest X-rays taken for other medical purposes eliminates exposure to additional radiation and allows screening without requiring additional medical procedures, specifically for examining bone density.

In recent years, deep learning, which is a machine learning technique that uses multilayer neural networks, has emerged as an effective technique for improving the performance of computer image recognition [16]. Subsequently, progress in orthopedics research has led to the use of deep learning models for osteoporosis screening [17]. In previous studies, diagnoses of osteoporosis based on radiographs of the lumbar spine and hip joint have been demonstrated [18,19], whereas the BMDs (g/cm^2^) of the hip and lumbar spine have been measured from radiographs of these sites [20,21]. Chest X-rays have also been used in two studies to diagnose osteoporosis [22,23]. However, the studies that used chest X-rays predicted only a “T-score less than −2.5” or “young adult mean (YAM) less than 80%.” The predictive performance indices obtained from these studies, in terms of the area under the curve (AUC), were 0.88 and 0.78, respectively, and prediction of the BMD (g/cm^2^), which is a continuous variable, was not performed. In addition, diagnosis (normal, osteopenia, or osteoporosis) could not be predicted based on the T-score using a single deep learning model. The deep learning models in past studies were trained on datasets that were each obtained from a single site, making it difficult to ensure the validity of the results because of the possibility of overtraining [24]. While a previous study on hip radiographs reported that ensemble learning of image data and patient clinical covariates can increase the prediction accuracy [25], there has been no report of such a learning method based on chest X-rays.

We hypothesized that a deep learning model can predict BMD using chest radiography. The purpose of this study was to develop a deep learning model trained on a large dataset collected from multiple institutions to predict BMD (g/cm^2^) and diagnosis based on the T-score (normal, osteopenia, and osteoporosis) using chest X-rays, age, and sex. By developing models with good predictive performance, we may be able to utilize chest X-rays as a screening tool for osteoporosis.

## 2. Materials and Methods

### 2.1. Patient Registration and Patient Data Collection

We conducted this retrospective multicenter study by collecting medical data from six hospitals in Japan (one university hospital and five general hospitals). This retrospective study was approved by the ethics committee of the lead hospital. This machine learning-based study was conducted in accordance with the Transparent Reporting of a Multivariable Prediction Model For Individual Prognosis Or Diagnosis (TRIPOD) guidelines [26] (Supplementary File S1).

The inclusion criteria were patients aged 20 years or older who visited any one of the facilities between April 2010 and July 2021, and underwent bone densitometry and chest X-ray imaging. The time gap between the bone densitometry examination and chest X-ray acquisition was within 6 months, in accordance with a previous study [20]. The dataset also included patients with implants or clinical features due to disease within the imaging range of the chest X-rays. The exclusion criteria were as follows: (i) patients whose chest X-rays did not include both lungs and clavicles, and (ii) patients whose chest X-rays were taken using portable equipment.

We extracted the anonymized image files from the image servers. All image files were in the “.dcm” format. The areal BMD was measured at the lumbar spine, femoral neck, and total hip using DXA. The details of the X-ray generator, image processing unit, image size, and DXA scanner at each facility are listed in Supplementary Table S1. We used the patient clinical covariates (age and sex), imaging data (chest X-rays), and results of bone densitometry (BMD and T-score from DXA) for the analysis in this study.

### 2.2. Data Preparation

We paired the bone densitometry and chest X-ray results of each patient. To improve predictability, we associated the age and sex with the chest X-rays and trained a deep learning model using ensemble learning [25]. We used the BMDs (g/cm^2^) measured at (i) the lumbar spine (average of L1–L4) and (ii) the lower of the values measured at the femoral neck and entire proximal femur [9]. The BMD values from the GE scanner were converted to Hologic values using the equations provided in Supplementary Table S2. For the T-score, we used the lowest value of the test results for the lumbar spine (average of L1–L4), femoral neck, and entire proximal femur [9]. We classified the participants into normal, osteopenia, and osteoporosis groups according to the World Health Organization (Geneva) (WHO) criteria [27]. The WHO defines normal as a T-score above −1.0, osteopenia as a T-score between −1.0 and −2.5, and osteoporosis as a T-score below −2.5. We labeled the BMD (g/cm^2^) and diagnosis based on the T-score (normal, osteopenia, osteoporosis) calculated against the chest X-rays.

### 2.3. Splitting the Dataset

**Figure 1:**
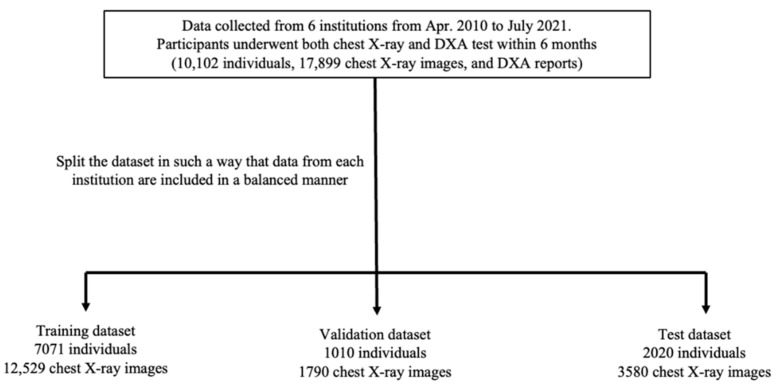
Dataset configuration. Note: DXA: dual-energy X-ray absorptiometry.

We randomly split the dataset collected from each hospital into training, validation, and testing datasets. We ensured that the data for each of the three labels (normal, osteopenia, and osteoporosis), in conjunction with their corresponding chest radiographs, age, and sex ratios, were randomly distributed in balanced numbers among the training, validation, and test datasets. The splitting ratios for the training, validation, and test datasets were 70%, 10%, and 20%, respectively. Figure 1 shows a flowchart of the dataset creation process, which ensured that the test dataset contained only new chest X-ray images that the model did not encounter during the training.

### 2.4. Image Preprocessing and Machine Learning

The specifications of the development environment were as follows: CPU: AMD EPYC 7452, GPU NVIDIA GTX TITAN X, Python 3.8.10, and PyTorch 1.10.0. To improve predictability, we performed data augmentation on the images extracted from the image server. For data augmentation, the image data were amplified via the application of ColorJitter (random brightness, contrast, saturation, and hue changes), RandomAffine (random geometric deformation), and RandomHorizontalFlip (random left-right flip) to each image. We then decomposed all chest X-rays into four (2 × 2) patches and resized them to 224 × 224 pixels. Each decomposed patch was vectorized and concatenated using ResNet50 [28]. These were then combined with the age and sex, and input into a three-layer perceptron with 128 hidden channels. The input batch size was 64 and optimization was performed using stochastic gradient descent. We trained the deep learning model as a regression for BMD and multiclass classification (one-vs.-all classification) for the T-score. In the multi-classification, we trained the deep learning model for three classification tasks as follows: (1) T-score above −1.0 vs. the rest; (2) T-score between −1.0 and −2.5 vs. the rest; and (3) T-score below −2.5 vs. the rest.

### 2.5. Statistical Analysis

The predictive performance of the deep learning model was evaluated using Scikit-Learn (https://scikit-learn.org/stable/; accessed on 1 July 2021). Data analysis was performed using a complete case dataset.

#### 2.5.1. Regression of BMD

We used the Pearson correlation coefficient (R-value), coefficient of determination (R-squared or R^2^), and mean absolute error (MAE) as the measures of performance in predicting BMD. In addition, a linear fitting curve and Bland–Altman plots were drawn. The R-value measures the linear correlation between the predicted value and ground truth, and considers only the sequential correlation, regardless of the absolute values. The linear fitting curve illustrates the overall direction of correspondence and modeling quality. The MAE is the error between the predicted values and standard references. Estimating the quality of the methods used for regression requires validation of the correlation between the measured values and reliable standards for accuracy, which is determined through the MAE and standard deviation of the MAE. The linear fitting curve illustrates the overall direction of correspondence and modeling quality. In the Bland–Altman plots, the error is plotted against the average value of a pair of predicted and true values.

#### 2.5.2. Classification of T-Score

The following metrics were used as a measure of performance in the classification of the T-score: (1) accuracy, (2) sensitivity, (3) specificity, and (4) AUC. The 95% confidence interval (CI) was also evaluated. The confusion matrix in this study was set as a 2 × 2 contingency table displaying the number of true positives, false positives, false negatives, and true negatives. The receiver operating characteristic (ROC) curve was created based on a plot of the true positive rate (sensitivity) against the false positive rate (1 − sensitivity).

## 3. Results

### 3.1. Patient Characteristics

The images were chest radiographs of 17,899 individuals (15,060 females and 2839 males, with ages ranging from 24 to 98 years (mean age 71.57 years)). From the chest radiographs, 3152 were categorized as normal results, 10,404 as osteopenia, and 4343 as osteoporosis based on DXA examination. Table 1 presents the baseline characteristics of the training, validation, and testing datasets.

### 3.2. Predictive Performance of Deep Learning Model

#### 3.2.1. Regression of BMD

**Figure 2:**
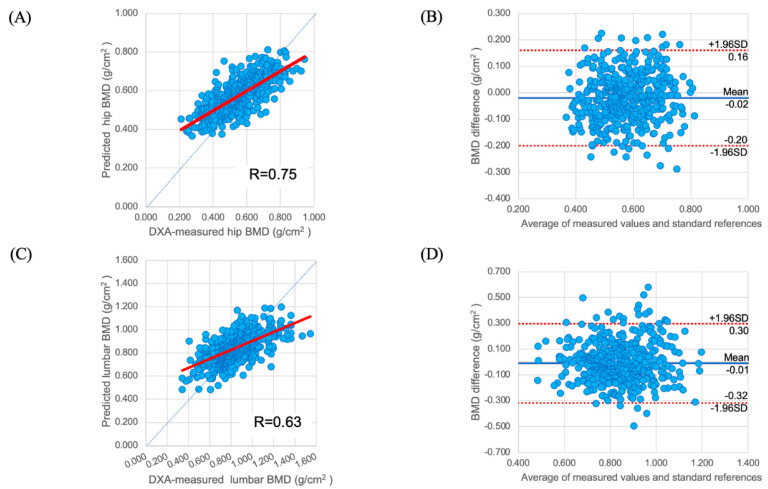
Predictive performance of regression on BMD. (**A**) Linear fitting curve and (**B**) Bland-Altman plot for the trained model for femoral BMD prediction. (**C**) Linear fitting curve and (**D**) Bland-Altman plot for the trained model for lumbar spine BMD prediction. Model predictions were compared with the ground truth. In the linear fitting curve, R is the Pearson correlation coefficient. Each point in the Bland-Altman plot represents a pair of DXA BMD and predicted BMD; the horizontal axis depicts the mean, whereas the vertical axis depicts the difference. Note: BMD: bone mineral density; DXA: dual-energy X-ray absorptiometry; SD: standard deviation.

The correlation plot and Bland–Altman plots for BMD predicted by the deep learning model and true BMD are shown in Figure 2. The predictive performance indices for femoral BMD are as follows: R-value of 0.75, R^2^ of 0.54, and MAE of 0.08. The predictive performance indices for lumbar spine BMD are as follows: R-value of 0.63, R2 of 0.40, and MAE of 0.12.

#### 3.2.2. Classification of T-Score

**Figure 3:**
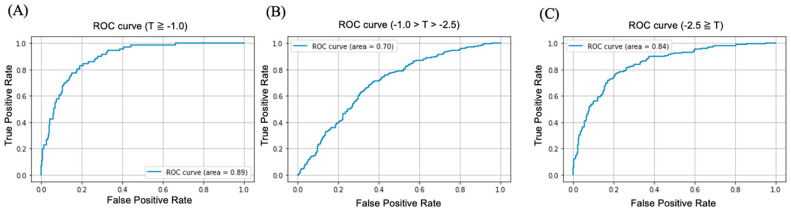
Receiver operating characteristic curve (ROC) for each class: (**A**) T-score greater than or equal to −1.0; (**B**) T-score between −1.0 and −2.5; (**C**) T-score less than or equal to −2.5.

The predictive performance of multiclass classification of the diagnoses based on the T-score (normal, osteopenia, and osteoporosis) is shown in Table 2. The ROC curves for the multiclass classification of the T-scores are shown in Figure 3. The predictive performance indices for diagnosis as normal (T-score above −1.0 vs. the rest) are an AUC of 0.89 (95% CI: 0.86–0.91), accuracy of 74.89% (95% CI: 71.21–77.45), sensitivity of 90.14% (95% CI: 87.35–92.41), and specificity of 72.24% (95% CI: 68.32–75.80). The predictive performance indices for diagnosis as osteopenia (T-score between −1.0 and −2.5 vs. the rest) are an AUC of 0.70 (95% CI: 0.68–0.72), accuracy of 66.06% (95% CI: 63.65–68.39), sensitivity of 71.28% (95% CI: 69.01–73.53), and specificity of 62.35% (95% CI: 59.94–64.77). The predictive performance indices for diagnosis as osteoporosis (T-score below −2.5 vs. the rest) are an AUC of 0.84 (95% CI: 0.82–0.86), accuracy of 77.83% (95% CI: 75.52–79.9), sensitivity of 77.27% (95% CI: 74.94–79.36), and specificity of 78.58% (95% CI: 76.32–80.55).

## 4. Discussion

In this study, we developed a deep learning model with ensemble learning based on chest X-rays, age, and sex to predict BMD (g/cm^2^) and diagnosis as per the T-score (normal, osteopenia, osteoporosis). With regard to the performance, the deep learning model could predict femoral BMD with R = 0.75, and predict “T-score = −1.0 or not” with an AUC of 0.89 and sensitivity of 90.14%. This study is the first to develop a deep learning model that predicts BMD (g/cm^2^) and T-scores using multiclass classification based on chest X-rays. The results demonstrated that the deep learning model may have potential for application in osteoporosis screening using chest X-rays in actual clinical practice.

The deep learning model was able to predict BMD using the chest X-rays. The predictive performance for hip BMD was R = 0.75, which indicates a high positive correlation with the true value [29]. Because none of the previous studies that predicted osteoporosis from chest X-rays were able to predict BMD, this study represents significant progress in this research area [22,23]. In comparison with the results of studies that predicted BMD from radiographs of the hip and lumbar spine using deep learning models [18,19], the results of our study were slightly inferior (previous studies: R = 0.81, 0.89; this study: R = 0.75). This may be due to the following reasons. (1) The site corresponding to the radiograph and the site where BMD was measured were different. (2) The training was performed based on setting the region of interest of the bone or dividing the image into sections instead of considering the entire image. Based on these factors, training the learning model such that the lumbar spine is cut out from the chest X-rays may improve the predictive performance. However, the performance of the method cannot be guaranteed. A previous study reported less accurate results in predicting the BMD of the lumbar spine than in predicting that of the hip [19]. Similarly, in this study, the predictive performance of the BMD differed between the hip and lumbar spine (hip: R = 0.75; lumbar spine: R = 0.63). The reason for this may be that, in comparison with those at the hip joint, the DXA measurements at the lumbar spine are subject to measurement errors due to osteoarthritis [30]. To address this problem, it is necessary to verify whether the performance can be improved through modifications of the labels and reorganization of the dataset.

The deep learning model was also able to predict diagnosis with moderate performance by utilizing T-scores with multiclass classification (normal, osteopenia, and osteoporosis) based on chest X-rays. The predictive performance indices were AUC = 0.89, 0.70, and 0.84, respectively. The predictive performance in the diagnoses of normal and osteopenia could not be compared because of the absence of similar studies in literature, but the predictive performance in diagnosis of osteoporosis was slightly inferior to that of a previous study [23]. Compared with previous studies that diagnosed osteoporosis using chest X-rays [22,23], our study has the following novel aspects: (1) a single deep learning model is classified into three classes: normal, osteopenia, and osteoporosis (multiclass classification); and (2) the T-score is used to predict diagnosis (normal or osteopenia). In screening for osteoporosis, it is important not only to identify the participants with T-scores below −2.5, but also those with T-scores between −1.0 and −2.5. This is because among the participants who underwent bone densitometry, the group diagnosed with osteoporosis had a higher fracture rate, whereas the group diagnosed with osteopenia had a higher number of patients. Therefore, the total number of fractures was higher in the group diagnosed with osteopenia than in the group diagnosed with osteoporosis [31]. Medical guidelines recommend further examination or therapeutic interventions for osteopenia [9,32,33]. Therefore, a deep learning model that can identify osteopenia is necessary. With regard to the predictive performance for T-score = −2.5, it was slightly lower in this study than in previous studies (Jang et al. [23]: AUC = 0.88; this study: AUC = 0.84). This was because in this study, data were collected from multiple centers, and thus a broad range of inclusion criteria was set. Large-scale and comprehensive data collection is necessary to ensure versatility. The previous study cited these factors as limitations, which were overcome in this study. The inferior performance indicates that there is potential for performance improvement. Previous studies have reported that learning based on setting regions of interest (shoulder, cervical and thoracic area, thoracic, and lumbar area) in chest X-rays improves the performance [23]. In the future, we will train our model using this approach.

Our deep learning model has the potential to perform osteoporosis screening using chest X-rays. In Japanese osteoporosis screening, a T-score below −1.0 indicates that the patient needs further examination. The predictive performance indices of the deep learning model developed in this study, with T-score = −1.0 as the cutoff, were sensitivity = 90.14% and specificity = 72.24%. From the viewpoint of triage screening for osteoporosis, high sensitivity (approximately 90%) and relatively low specificity (approximately 40–60%) are considered acceptable for clinical decision rules [34]. Therefore, we can use this deep learning model to screen for osteoporosis. In Japan, 40 million people over the age of 40 are screened for lung cancer using chest X-rays [35]. By applying the deep learning model to these potential participants to screen for osteoporosis, we could find five million new osteoporosis patients based on the age range of the examinees and age-specific incidence of osteoporosis [35,36]. Appropriate therapeutic interventions for these patients would then help prevent fragility fractures [37].

The strength of this study lies in the collection of diverse data from multiple institutions. The advantages of multicenter studies are (i) the ability to prevent overfitting by collecting a large amount of data [24] and (ii) the ability to conduct comprehensive research by using data obtained from different conditions and environments, thereby allowing medical research to be conducted in clinical settings [38]. In this study, we collected approximately 18,000 training data points from approximately 10,000 cases, which included almost all chest X-ray images taken at multiple institutions in Japan and with various medical devices over a long duration. This allowed for diverse patient datasets (images that included implants and clinical features due to disease) collected from multiple examination settings, including X-ray generators, image processing units, and DXA scanners. This supports this study’s validity as an epidemiological study and ensures its internal validity. On the contrary, to be used in clinical practice, external validity must be assessed using data from other institutions.

However, this study has several limitations. First, we did not develop multiple trained models or validate their predictive performance. Transfer learning using pretrained models is common in deep learning [39]. A previous study evaluated various learned models and reported differences in their performance [20]. In this study, we used ResNet50 because of its short processing time [28]. In the future, training with different learning models may lead to improved performance. Second, we considered only age and sex as the patient variables in predicting the BMD and T-scores. However, various patient factors can influence the incidence of osteoporotic fractures [40]. In this study, we trained our deep learning model using chest X-rays, age, and sex. This was because we believed that learning from the information contained in the image file (image, age, and sex) would not change the current workflow in an actual clinical setting. However, a previous study reported that training a deep learning model with patient clinical covariates, such as height, weight, and fracture history, improved the performance [25]. Further, various diseases (COPD, rheumatism, etc.) that coexist with osteoporosis should be considered [41,42]. Considering this, we can train our deep learning model with these factors to verify the possibility of improving the performance. Third, we have not evaluated the predictive accuracy of the developed training model for each age group (young, middle-aged, and older adults). Osteoporosis is prevalent in aged women, and this population group is the target for screening [9]. Secondary analysis for this age group is required to make the analysis more relevant to actual clinical practice. Fourth, we did not perform an external validation. Most studies on deep learning models have not evaluated the validity of the models in different environments [38]. Although this study prepared a dataset with data collected from multiple facilities, we were unable to validate our model using data from entirely different clinical settings. To train our deep learning model as a programmed medical tool, it is necessary to evaluate the predictive performance using data collected at different facilities and from different racial groups. Fifth, while the deep learning model could diagnose osteoporosis on guidelines based on T-score, this did not necessarily imply that it could understand the pathophysiology of osteoporosis, including causative disease and comorbidities. We developed this deep learning model using radiographs, bone densitometry, age, and gender but did not consider medical history such as comorbidities. Therefore, to confirm whether the results of this deep learning model analysis are normal, the physician should interview and examine the patient, perform blood tests, and make a definitive diagnosis using DXA.

## 5. Conclusions

We developed a deep learning model based on ensemble learning of chest X-rays, age, and sex to predict BMD (g/cm^2^) and diagnosis according to the T-score (normal, osteopenia, osteoporosis). With this model, chest X-rays taken for various medical reasons can be used to identify patients at risk for osteoporosis without additional radiation exposure or cost, and without the possibility of behavioral changes in the examinee. This may improve screening for osteoporosis. To realize the goal of clinical application, we need to further improve the predictive performance and validity of the deep learning model.

## 6. Patents

A patent application for the results of this study has been filed in Japan (No. 21ZP324).

## Figures and Tables

**Table 1:** Demographic characteristics of the dataset.

		Training Dataset	Validation Dataset	Test Dataset	Overall
Participant		12,529	1790	3580	17,899
age (years), mean ± SD		71.94 ± 10.05	71.24 ± 10.94	71.54 ± 11.27	71.57 ± 10.75
Sex	Female (%)	10,544 (84.16%)	1508 (84.25%)	3008 (84.02%)	15,060 (84.14%)
	Male (%)	1985 (15.84%)	282 (15.75%)	572 (15.98%)	2839 (15.86%)
BMD (g/cm^2^), mean ± SD	Lumbar	0.88 ± 0.19	0.89 ± 0.21	0.88 ± 0.20	0.88 ± 0.20
	Hip	0.58 ± 0.12	0.59 ± 0.15	0.58 ± 0.13	0.58 ± 0.13
T-score mean ± SD	Lumbar	−1.51 ± 1.56	−1.53 ± 1.68	−1.51 ± 1.60	−1.52 ± 1.61
	Hip	−2.145 ± 1.17	−2.15 ± 1.40	−2.16 ± 1.10	−2.15 ± 1.22
T-score categories, *n* (%)	Normal	2204 (17.59%)	317 (17.71%)	631 (17.63%)	3152 (17.61%)
	Osteopenia	7287 (58.16%)	1038 (57.99%)	2079 (58.07%)	10,404 (58.13%)
	Osteoporosis	3038 (24.25%)	435 (24.30%)	870 (24.30%)	4343 (24.26%)

Note: BMD: bone mineral density; SD: standard deviation.

**Table 2:** Performance metrics of the model for the test dataset. The accuracy, sensitivity, specificity, and AUC in the respective ranges, with T-scores of −1.0 and −2.5 as cutoffs, are shown.

	AUC (95% CI)	Accuracy (%) (95% CI)	Sensitivity (%) (95% CI)	Specificity (%) (95% CI)
T-score ≥ −1.0	0.89 (0.86–0.91)	74.89 (71.21–77.45)	90.14 (87.35–92.41)	72.24 (68.32–75.80)
−1.0 > T-score > −2.5	0.70 (0.68–0.72)	66.06 (63.65–68.39)	71.28 (69.01–73.53)	62.35 (59.94–64.77)
−2.5 ≥ T-score	0.84 (0.82–0.86)	76.47 (75.52–79.90)	81.25 (74.94–79.36)	73.68 (76.32–80.65)

Note: AUC: area under the curve; CI: confidence interval.

## Data Availability

The data that support the findings of this study are available upon request from the corresponding author. The data are not publicly available because of privacy or ethical restrictions.

## Supplementary Materials

The following supporting information can be downloaded at: https://www.mdpi.com/article/10.3390/biomedicines10092323/s1, Table S1: title X-ray generator, image processing unit, image size, and DXA scanner at each facility; Table S2: title Equations for converting BMD value of GE to that of Hologic DXA scanners.
