# Supplementary material for: Deep Learning for Bone Mineral Density and T-Score Prediction from Chest X-rays: A Multicenter Study
Source: Biomedicines. 2022 Sep 19;10(9):2323. doi: 10.3390/biomedicines10092323 (PMC9496220; doi:10.3390/biomedicines10092323)
Supplement: Supplementary file 1 [file biomedicines-10-02323-s001.zip › biomedicines-1916522-supplementary.pdf]

Supplementary Table S1. X-ray generator, image processing unit, image size, and DXA scanner at each facility.

	JCHO Tokyo Shinjuku Medical Center	Kakogawa Medical Center	Miyamoto Orthopaedic Surgeon Clinic	The Jikei University Kashiwa Hospital	Nagoya Medical Center	Gamagori City Hospital
X-ray generator	RAD Speed Pro (Shimazu)	UD150L-40 (Shimazu)	DREX-PZ10 (Toshiba)	BENEO-Fx (Fujifilm Medical)	RAD Speed Pro (SHIMAZU)	Model TF-6TL-6 (Toshiba)
Image processing unit	CALNEO GL, CALNEO Flow (Fujifilm Medical)	Console Advance (Fujifilm Medical)	CS-7 (Konica Minolta)	CALNEO Smart G77 (Fujifilm Medical)	CS-7 (Konica Minolta)	CALNEO Smart C12 (Fujifilm)
Image size	2373×2373 pixel	2140 × 1760 pixel	4020 × 4020 pixel	1760 × 1760 pixel	2430 × 1994 pixel	4,892×4,020 pixel
DXA scanner	Discovery C (Hologic)	Horizon X (Hologic)	Explorer (Hologic)	Lunar Prodigy (GE Healthcare)	Discovery A (Hologic)	Discovery (Hologic)

Note: DXA: Dual-Energy X-Ray Absorptiometry.

Supplementary Table S2. Equations for converting BMD value of GE to that of Hologic DXA scanners.

Measurement site	Conversion equation
Lumbar spine	Hologic BMD = 0.918 × GE BMD - 0.038
Hip	Hologic BMD = 0.971 × GE BMD - 0.037

Note: BMD: Bone mineral density.
